# Light Quality as a Non-Chemical Elicitor of Polyphenol Accumulation in Rosemary (*Salvia rosmarinus* Spenn.) Cell Suspension Cultures

**DOI:** 10.3390/plants15213322

**Published:** 2026-10-27

**Authors:** Gustavo J. Cáceres-Cevallos, Almudena Bayo-Canha, María Quílez, María J. Jordán

**Affiliations:** Research Group on Rainfed Agriculture for Rural Development, Department of Rural Development, Oenology and Sustainable Agriculture, Murcian Institute of Agricultural and Environmental Research (IMIDA), La Alberca de las Torres, 30150 Murcia, Spain; almudena.bayo@carm.es (A.B.-C.); maria.quilez@carm.es (M.Q.); mariaj.jordan@carm.es (M.J.J.)

**Keywords:** *Salvia rosmarinus*, suspension cells, physical elicitation, LED light, rosmarinic acid, antioxidant activity

## Abstract

*Salvia rosmarinus* Spenn. is a valuable source of phenolic compounds, but their production is affected by environmental and seasonal variability. Addressing these limitations, plant cell suspension cultures provide a controlled platform for secondary metabolite production, with elicitation offering a strategy to enhance metabolite accumulation and biosynthetic efficiency. In this context, our study investigated the effects of LED light as a physical elicitor on the growth and phenolic profile of rosemary cell suspension cultures. Suspension cells were exposed to five different light conditions: darkness, white light (WL), a mixture of blue, red, and far-red light (B+R+FR), blue light (B) and red combined with far-red light (R+FR) over 2 weeks. Growth parameters, phenolic profile, and antioxidant activity were evaluated. We found that, while B produced the highest packed cell volume, the R+FR combination significantly enhanced biomass accumulation. Regarding phenolic content, the B+R+FR treatment led to the highest production of salvianic and rosmarinic acids. This combination of wavelengths showed a strong correlation with improved antioxidant capacity. These findings suggest that tailored LED spectra, particularly the B+R+FR combination, have good potential to improve the phytochemical quality of rosemary in vitro, supporting the development of a sustainable platform for the production of high-value bioactive compounds.

## 1. Introduction

*Salvia rosmarinus* Spenn. (syn. *Rosmarinus officinalis* L.), a perennial shrub of the Lamiaceae family, is one of the most commercially and therapeutically significant Mediterranean aromatic medicinal plants [1]. The shrub’s industrial value primarily stems from its high concentrations of non-volatile bioactive phenolic compounds. Among these, the phenylpropanoid pathway yields a rich array of polar hydroxycinnamic acid derivatives, predominantly rosmarinic acid, accompanied by salvianic acid (danshensu), caffeic acid, chlorogenic acid, and feruloylquinic acids, alongside lipophilic abietane diterpenes such as carnosic acid and carnosol [2].

These metabolites exhibit potent antioxidant, anti-inflammatory, and antimicrobial properties, explaining their extensive use in traditional medicine and the pharmaceutical industry [3]. Among plant-derived antioxidant extracts, rosemary extract is recognized as a food additive in the European Union (E392). Under EU legislation, rosemary extracts are authorized for use as antioxidants in certain food categories. The main antioxidative compounds in these extracts are carnosic acid and carnosol, as noted in Commission Regulation (EU) No 231/2012 and Regulation (EC) No 1333/2008 [4]. This regulatory approval, combined with the well-documented antioxidant activity of rosemary-derived compounds, underscores the potential strategic significance of rosemary extracts for use in the food and nutraceutical industries [5].

However, the production of these secondary metabolites from wild-harvested or conventionally cultivated rosemary is often compromised by environmental variability, seasonal fluctuations, and geographical constraints, which limit the consistency and quality of the chemical profile [6,7]. To overcome these challenges, plant cell suspension cultures have emerged as a sustainable and scalable biotechnological platform. This system allows for the biosynthesis of specialized metabolites in highly controlled, sterile environments, ensuring year-round productivity independent of external climatic factors [8]. Its effectiveness has already been demonstrated in rosemary cell cultures, particularly for the production of phenolic acids and diterpenes with well-established antioxidant properties. Early studies demonstrated the accumulation of rosmarinic acid in rosemary callus, with culture conditions such as basal medium and auxin supplementation influencing both biomass production and metabolite accumulation [9]. Carnosic acid has also been detected in green rosemary callus, whereas its accumulation was not observed in non-green callus maintained under dark conditions, suggesting an important role for tissue differentiation and light availability in diterpenoid biosynthesis [10]. In addition, rosemary callus has been shown to produce volatile monoterpenes, including α-pinene, β-pinene, and camphor, demonstrating that in vitro cultures can serve as controlled systems for the production of diverse classes of secondary metabolites [11].

As stated above, this approach can provide a consistent supply of high-quality and high-value secondary metabolites, while their production can be further enhanced by manipulating culture conditions with elicitation strategies [12]. An elicitor is defined as an agent that, when added at low concentrations, triggers a plant’s defense mechanisms and redirects metabolic flux toward the hyper-accumulation of secondary metabolites [13]. These are generally classified into biotic elicitors, derived from biological sources like polysaccharides or fungi, and abiotic elicitors, physical or chemical factors such as heavy metals or radiation [14]. In this context, elicitation strategies have demonstrated considerable potential for enhancing the accumulation of bioactive compounds in rosemary cultures. For instance, the combined application of UV-B radiation and melatonin has been shown to increase total phenolic content and promote rosmarinic acid accumulation in rosemary callus cultures [15]. These findings highlight the potential of controlled environmental stimuli, particularly light-based treatments, to modulate secondary metabolism and enhance the production of bioactive compounds.

Among abiotic strategies, light-emitting diodes (LEDs) have revolutionized in vitro cultivation as a high-precision physical elicitor [16]. This technology provides significant advantages over traditional fluorescent lighting, including the ability to emit specific monochromatic wavelengths, high energy efficiency, and minimal heat emission [17]. Furthermore, optimized LED spectra can simultaneously promote nutrient uptake, enhance biomass growth, and encourage the hyperaccumulation of targeted specialized metabolites, all without triggering the severe growth-defense trade-offs associated with chemical stress [6,18].

In this regard, light elicitation has been associated with the response to light quality of specific plant photoreceptors, such as phytochromes and cryptochromes, which may modulate gene expression in the phenylpropanoid and flavonoid biosynthetic pathways [19]. Specifically, the application of different light spectra has effectively increased metabolite yields in several Lamiaceae species, including *Ocimum basilicum* [20], *Prunella vulgaris* [21], and *Scutellaria lateriflora* [22].

Despite recent advances, the effects of various monochromatic and mixed LED light spectra on rosemary cell suspension cultures are still not well understood. Therefore, testing different LED lighting regimes could yield important insights into how light quality affects biomass accumulation, specific phenolic profiles, and the antioxidant potential of the resulting cell extracts. In this context, we aimed to evaluate the impact of different LED light spectra on growth performance and phenolic accumulation in *S. rosmarinus* cell cultures, while also assessing the bioactivity of the extracts as a sustainable source of high-value antioxidants.

## 2. Results

### 2.1. Growth Parameters

The growth parameters assessed differed between the treatments, as shown in Table 1. The packed cell volume (PCV) was highest under monochromatic blue light (B), followed by the combination of red and far-red light (R+FR). In contrast, B resulted in low cell biomass per liter, even lower than the dark treatment used as a control. On the other hand, the R+FR treatment was associated with significantly higher biomass.

Regarding pH, the values ranged from 5.7 to 6.4, with the lowest value observed with the dark treatment. Brix levels (°Bx) decreased from a baseline value of 4.3 ± 0.2 °Bx (based on measurements taken in the initial culture conditions, see Table S1) to final values ranging from 0.8 ± 0.3 to 1.1 ± 0.1 °Bx across the treatments.

### 2.2. Phenolic Profile

The high-performance liquid chromatography (HPLC)-diode array detector (DAD) analysis identified five major phenolic compounds (Figure 1). Salvianic acid content significantly increased under B and the combined spectra R+FR and B+R+FR (Figure 1a).

Regarding chlorogenic acid, B induced the highest concentrations of this compound (Figure 1b). In contrast, the R+FR treatment promoted greater accumulation of caffeic acid and 4-*O*-feruloylquinic acid (Figure 1c,d). Rosmarinic acid was the predominant phenolic compound detected in the cell suspension cultures (Figure 1e), with the highest levels observed under B and B+R+FR treatments.

### 2.3. Antioxidant Activity

Antioxidant activity was evaluated using ferric reducing antioxidant power (FRAP), 2,2-diphenyl-1-picrylhydrazyl (DPPH^•^), and 2,2′-azino-bis (3-ethylbenzothiazoline-6-sulfonic acid (ABTS^•+^) assays (Figure 2). In general, treatments containing blue wavelengths (B and B+R+FR) significantly enhanced antioxidant capacity compared to that under dark and WL conditions. The highest FRAP and DPPH values were observed under B and B+R+FR treatments, while ABTS^•+^ activity peaked under the B+R+FR spectrum. In contrast, dark and WL treatments were associated with the lowest antioxidant responses.

Pearson’s correlation analysis (Figure 3) revealed strong positive correlations between antioxidant activity and the accumulation of several phenolic compounds, particularly rosmarinic and salvianic acids. FRAP and DPPH^•^ assays showed especially high correlation coefficients with these metabolites, reflecting their major contribution to the antioxidant capacity of the extracts.

## 3. Discussion

The exposure of rosemary cell suspension cultures to different LED light spectra showed that phenolic accumulation can be modulated without necessarily affecting culture growth.

While all light treatments increased the levels of phenolic compounds compared to those in the control group, some cultures exhibited reduced growth, particularly under WL and monochromatic blue (B) conditions. In particular, while the highest PCV was obtained with B, it produced low biomass. This reduction may be associated with the higher energy of blue photons, which has been reported to induce oxidative stress and stimulate secondary metabolism in some in vitro plant systems [6]. Therefore, the high PCV under B may reflect changes in cell hydration, aggregation, or morphology rather than a proportional increase in dry biomass.

Interestingly, despite the lower growth observed under WL and B conditions, sucrose consumption remained similar to that observed in the control, indicating sustained metabolic activity [23]. Additionally, all light treatments resulted in increased pH levels in the culture medium, which may be associated with active nutrient uptake and metabolic activity. Specifically, Lim et al. [7] suggested that changes in medium pH can be related to nitrogen uptake, which may indirectly affect secondary metabolism.

Qualitative and quantitative profiling of the *Salvia rosmarinus* cell suspension extracts by HPLC-DAD analysis identified five major phenolic compounds: salvianic, chlorogenic, caffeic, 4-*O*-feruloylquinic, and rosmarinic acids. The accumulation levels of these metabolites were found to be highly sensitive to the cultivation environment, specifically the spectra of the LED light [19].

Regarding compound accumulation, the B+R+FR treatment significantly increased the levels of rosmarinic acid compared to the R+FR treatment. Meanwhile, salvianic acid accumulation was similarly high in both the B+R+FR and R+FR treatments. The concentrations of rosmarinic acid observed under the B+R+FR treatment reached values within the typical range of 15–50 mg g^−1^ DW, which has been reported in some in vitro Lamiaceae cultures, including *Salvia bulleyana* [24], *Salvia atropatana* [25], and *Agastache rugosa* [26]. Rosmarinic acid is the primary contributor to the polar antioxidant pool and serves as the main structural factor for the bioactivity of the extracts.

Studies within the Lamiaceae family have shown that strategically manipulating both monochromatic and mixed light conditions can effectively stimulate the biosynthetic pathways involved in phenylpropanoid and hydroxycinnamic acid production [6,19,20]. This manipulation can alter the concentration of specific compounds depending on the wavelengths detected by photoreceptor cells. In this regard, the varying accumulation of salvianic, rosmarinic, chlorogenic, and caffeic acids under different light spectra suggests that light quality influences the distribution of phenolic metabolites in rosemary cell suspension cultures.

Specifically, B seems to increase the accumulation of salvianic, chlorogenic, and rosmarinic acids, which is consistent with previous studies in Lamiaceae species in which monochromatic blue light has been associated with higher phenolic acid levels [21]. For instance, in *Ocimum basilicum*, blue wavelengths have been identified as the most effective range for enhancing the production of rosmarinic acid and eugenol [3,20]. A similar effect was observed in *Prunella vulgaris*, where exposure to blue wavelengths maximized total phenolic and flavonoid content [21].

This response could be related to the activation of blue light photoreceptors, such as cryptochromes and phototropins, which have been reported to participate in signaling cascades associated with phenylpropanoid metabolism and phenylalanine ammonia-lyase enzyme (PAL) regulation [18]. PAL is a key gateway enzyme of the phenylpropanoid pathway and is closely linked to the biosynthesis of hydroxycinnamic acid derivatives, including rosmarinic acid [14]. Additionally, Krzemińska et al. [24] indicated that this spectrum increased oxidative stress levels and limited biomass growth in sensitive species such as *Salvia bulleyana*. A similar response could explain the increased accumulation of antioxidant phenolic compounds observed in our study.

In the case of the red and far-red treatment (R+FR), the higher accumulation of salvianic, caffeic, and 4-*O*-feruloylquinic acids suggests that this spectral combination may influence phenolic metabolism differently from B. Studies in species such as *Artemisia annua* [7] and *Stevia rebaudiana* [17] have shown that R+FR can modulate the phytochrome photoequilibrium (active-to-total phytochrome ratio), a process that has been associated with changes in morphogenesis and secondary metabolism. Additionally, in *Eucomis autumnalis*, specific LED light spectra altered the concentrations of individual phenolic acids, indicating that red light perception by phytochromes can prioritize certain branches of the hydroxycinnamic acid pathway [19].

It is also important to note that under R+FR conditions, the increase in caffeic acid content, an immediate precursor of rosmarinic acid [27], might suggest a partial accumulation of upstream intermediates in the rosmarinic acid biosynthetic route. However, the higher caffeic acid levels were not accompanied by a proportional increase in rosmarinic acid accumulation (Figure 1c,e).

Exposure to blue, red, and far-red wavelengths (B+R+FR) resulted in a more balanced physiological response. According to Cioć et al. [19], the simultaneous perception of blue and red/far-red wavelengths by different photoreceptor systems may generate interactive effects that differ from those produced by monochromatic light. Further, Murthy et al. [14] noted in their review that combining photoreceptor stimulation can activate key transcription factors such as HY5, which operates alongside the COP1 module at the core of the light-signaling network. As a result, it can directly regulate the expression of key enzymes involved in phenylpropanoid biosynthesis, thereby driving the production of phenolic compounds.

These different metabolic outcomes can be explained by photoreceptor-specific signal transduction pathways. Considering this, it has been recognized that monochromatic B light triggers cryptochromes (CRY1/CRY2) and phototropins, stabilizing the HY5 transcription factor to upregulate gateway phenylpropanoid enzymes (such as PAL and TAT) while inducing mild photo-oxidative stress [14,19]. On the other hand, the R+FR treatment modulates phytochrome photo-equilibrium (Pfr/Pr ratio), which could enhance early precursor synthesis (such as caffeic and 4-*O*-feruloylquinic acids) but lacks the blue-light signaling necessary for maximal downstream conversion into rosmarinic acid [20]. The combined B+R+FR spectrum enables simultaneous perception across CRY and PHY systems, triggering photoreceptor crosstalk that optimizes flux through both the phenylpropanoid and tyrosine-derived branches, thereby maximizing overall phenolic accumulation [24].

Our findings align with those of Krzemińska et al. [24] in *Salvia bulleyana*, where a specific spectral combination resulted in the highest levels of rosmarinic acid and total phenolic content. Similarly, our results agree with the study by Cioć et al. [19] in *Eucomis autumnalis*, which demonstrated that a spectrum enriched with far-red light enhanced the simultaneous production of phenolic acids and flavonoids. In contrast, rosmarinic acid levels in *Salvia atropatana* were lower after exposure to a combination of red and blue light (at a ratio of 7:3) than to monochromatic light [25]. This difference indicates that the effects of light quality on in vitro plant cultures are species-dependent.

Taken together, these findings highlight the potential of light-spectrum modulation as an effective strategy for enhancing secondary metabolism in rosemary cell cultures. Previous studies from our laboratory demonstrated that chemical elicitation with methyl jasmonate (MeJA) promotes the accumulation of phenolic compounds [28]. Nevertheless, the concentration of key metabolites, particularly rosmarinic acid, was lower under the optimal MeJA treatment (75 µM) than under the B and combined B+R+FR treatments, suggesting that spectral modulation may provide a more effective stimulus for the accumulation of specific phenolic compounds.

To assess the bioactive potential of *S. rosmarinus* cell extracts, we conducted three complementary spectrophotometric antioxidant assays (DPPH^•^, FRAP, and ABTS^•+^), it being important to use multiple methods, since they operate through distinct chemical mechanisms.

The high antioxidant activity observed under the combined B+R+FR treatment, as well as the monochromatic B treatment, was associated with higher accumulation of hydroxycinnamic acid derivatives. Our data revealed a highly significant positive correlation between antioxidant capacity and the levels of caffeic, salvianic, and rosmarinic acids, suggesting that these phenolic compounds may contribute substantially to the antioxidant capacity of the extracts. Additionally, it is noteworthy that the highest synthesis levels of salvianic and rosmarinic acids occurred under the B+R+FR spectrum. This pattern suggests a possible interactive effect among wavelengths, potentially related to the simultaneous perception of blue, red, and far-red light by different photoreceptor systems.

Interestingly, while R+FR promoted the highest levels of caffeic and 4-*O*-feruloylquinic acids, it did not produce the highest antioxidant activity. This is likely due to the B+R+FR treatment resulting in greater levels of salvianic and rosmarinic acids. Thus, although red/far-red light perception by phytochromes may be associated with the accumulation of specific precursors [19], the inclusion of blue wavelengths in the B+R+FR treatment may favor the accumulation of phenolic compounds that contribute more to antioxidant capacity. Nevertheless, the strong correlation between caffeic acid and all antioxidant assays highlights its significant contribution to overall antioxidant activity, despite its lower total abundance.

In this context, while R+FR spectra increased the levels of phenolic compounds (specifically caffeic and 4-*O*-feruloylquinic acids) this was not reflected in antioxidant capacity as measured by DPPH^•^, FRAP, and ABTS^•+^ assays. This suggests that the bioactivity of the extracts depends more on the individual chemical structures and molar reactivities of the predominant metabolites, rather than just the total mass of phenolics [2]. Furthermore, treatments with blue light (B and B+R+FR) specifically enhanced rosmarinic acid levels, which is the main contributor to the polar antioxidant activity in rosemary extracts [3].

It is also important to understand that different antioxidant assays operate through distinct chemical mechanisms: DPPH assesses hydrogen atom transfer in organic environments, while FRAP evaluates single-electron reducing power concerning ferric ions. Therefore, the superior antioxidant performance observed under B+R+FR and B spectra reflects a qualitatively enriched phenolic matrix, particularly in rosmarinic and salvianic acids, rather than simply the abundance of precursor compounds.

Overall, the wavelength-dependent differences observed in phenolic accumulation and antioxidant capacity indicate that rosemary cell suspension cultures respond distinctly to specific LED spectra. The B+R+FR treatment produced the most favorable biochemical response, suggesting that the combination of blue, red, and far-red wavelengths may promote a more balanced interaction between growth-related processes and hydroxycinnamic acid accumulation.

Although future studies including gene expression analysis, enzymatic activity, and oxidative stress markers are needed to provide deeper insight into the molecular mechanisms involved, the results of this study indicate that LED spectral composition can be strategically adjusted to improve the phytochemical performance of rosemary cell suspension cultures.

## 4. Materials and Methods

### 4.1. Plant Material and Callus Establishment

To establish the callus culture, we used leaves from a pre-selected rosemary plant, which had been obtained through in vitro methods. The explants were cultured in Murashige & Skoog medium enriched with vitamins, supplemented with 2% sucrose, and growth regulators: kinetin (0.465 µM) and indol-3-acetic acid (0.571 µM) (all medium components were sourced from Duchefa Biochemie, Haarlem, The Netherlands). This process was repeated five times to obtain a homogeneous callus every 3 weeks in dark conditions at 25 °C.

### 4.2. Cell Line Cultivation

Suspension cultures were initiated by inoculating selected white friable calli derived from rosemary callus at a concentration of 1% (*w*/*v*) in Murashige & Skoog liquid medium, which was enriched with 3% sucrose and the same growth regulators as those mentioned in Section 4.1. The cells were cultivated at 25 °C in the dark while being shaken at 110 rpm. To establish a uniform line (Figure S1), successive subcultures were performed by diluting the culture at a ratio of 1:5 every 15 days for 3 months. Additionally, growth parameters were evaluated to determine the lag, exponential, and stationary phases of the culture (see Figure S2).

### 4.3. Elicitation Treatments

Rosemary cell suspension cultures cultivated in the dark were transferred at a ratio of 1:5 (*v*/*v*) by mixing 20 mL of the culture with 80 mL of the MS media mentioned above in 250 mL flasks. The flasks were then randomly assigned to one of five light treatments (Figure 4): darkness (used as the control), broad-spectrum white light (WL, 400–700 nm), monochromatic blue light (B, λ_max_ = 450 nm, range 400–500 nm), a red/far-red combination (R+FR, red: λ_max_ = 660 nm, 600–700 nm; far-red: λ_max_ = 730 nm, 700–800 nm) and a blue/red/far-red combination (B+R+FR). The spectral ratios used for the combined light treatments were 25:40:35 for B+R+FR and 55:45 for R+FR. The photon flux density at culture level was maintained at 45 ± 5 µmol m^−2^ s^−1^ and measured using a spectral PAR meter (PG200N, UPRtek, Miaoli County, Taiwan). Chamber temperature was maintained at 24 ± 1 °C under a 16 h light/8 h dark photoperiod.

The LED spectral treatments were chosen based on our recent systematic review of Lamiaceae photomorphogenesis [29]. To modulate the phytochrome photo-equilibrium (Pfr/Pr) and stimulate morphogenesis and precursor pathways, we applied an R+FR ratio of 55:45. Additionally, the combination of B+R+FR light with a ratio of 25:40:35 includes a moderate amount of B light (25%). This B light activates cryptochrome-mediated secondary metabolism, while the balanced R and FR background (40:35) facilitates crosstalk between dual photoreceptors to optimize the accumulation of secondary metabolites.

For each light treatment, three independent biological replicates were used. Cultures were maintained under the corresponding light conditions for 2 weeks before growth, phenolic profile, and antioxidant activity analyses.

### 4.4. Growth Parameters Measurement

#### Packed Cell Volume, pH, Brix Levels and Biomass

The PCV was obtained by centrifuging 10 mL of cell suspension for 10 min at 2000× *g*. The supernatant from each sample was then used to determine the Brix levels with a refractometer (ORA 10BA, KERN & SOHN, Balingen, Germany) and the pH with a pH meter (HI2003, Hanna Instruments Deutschland GmbH, Vöhringen, Germany).

Biomass was assessed by measuring the dry weight (DW) of the cell suspension. Aliquots of 20 mL were filtered under vacuum using a Kitasato flask. The retained cells were dried in an oven at 65 °C for 24 h (having confirmed that this was sufficient to reach constant weight). The results were expressed as biomass concentration in grams per liter (g L^−1^).

### 4.5. Phenolic Profile Determination

#### 4.5.1. Phenolic Compounds Extraction

Phenolic compounds extraction was adapted from the methodology described by Cáceres-Cevallos et al. [30]. Briefly, 250 mg of lyophilized cells were combined with 5 mL of an extraction solution made up of dimethyl sulfoxide and 1% acidified water in a 1:1 ratio. The samples were then agitated using a rotary shaker at 20 °C in an inert atmosphere for 1 h. After the extraction process, the samples were centrifuged at 4 °C and 2347× *g* for 10 min. The supernatant was filtered through a 0.45 µm filter and stored at −20 °C until HPLC analysis and antioxidant activity assays.

#### 4.5.2. HPLC-DAD Identification and Quantification

The HPLC analyses were performed using an Agilent 1200 series system (Agilent, Waldbronn, Germany) equipped with a DAD and a C18 column with chromatographic conditions adapted from our previous work [31]. The mobile phase consisted of acetonitrile and acidified water (0.05% formic acid), and analytes were separated by gradient elution at a flow rate of 1.0 mL/min. Chromatograms were acquired at 280, 306, and 330 nm. Phenolic compounds were identified and quantified using the standards listed in Table S2, expressed as mg g^−1^ dry weight extract. All standards were purchased from Sigma-Aldrich (Merck, Darmstadt, Germany).

For the main phenolic compounds quantified, the limits of detection (LOD) and limits of quantification (LOQ) for rosmarinic acid were 1.6 μg/mL and 5.4 μg/mL, respectively. For caffeic acid, the LOD and LOQ were 0.9 μg/mL and 3.1 μg/mL, respectively.

### 4.6. Antioxidant Activity Evaluation

#### 4.6.1. ABTS^•+^ Assay

ABTS^•+^ radical-scavenging activity was evaluated following the method reported by Quílez et al. [32], with minor modifications. The ABTS^•+^ radical solution was prepared by reaction of ABTS^•+^ with potassium persulfate and allowed to stand in the dark for 12–16 h. Before use, it was diluted in 95% ethanol to an absorbance of 0.70 ± 0.02 at 734 nm and equilibrated at 30 °C. Sample aliquots (15 µL) were mixed with 1.5 mL of ABTS^•+^ solution, incubated in the dark for 3 min, and absorbance was measured at 734 nm. Quantification was performed using Trolox as a standard, and results were expressed as M of Trolox equivalents g^−1^ of extract.

#### 4.6.2. DPPH^•^ Assay

DPPH^•^ radical-scavenging activity was determined following the method of Farhat et al. [33]. Briefly, 500 µL of extract were mixed with 1 mL of 0.1 mM DPPH^•^ solution in methanol. The mixture was incubated for 20 min at room temperature in the dark, and absorbance was recorded at 517 nm against a blank containing 500 µL of extract and 1 mL of methanol. Results were expressed as µmol Trolox equivalents g^−1^ of extract.

#### 4.6.3. FRAP Assay

The FRAP assay was performed according to a modified procedure described by Szabó et al. [34]. The FRAP reagent was prepared by mixing acetate buffer (300 mM, pH 3.6), 10 mM 2,4,6-tripyridyl-s-triazine (TPTZ) in 40 mM HCl, and 20 mM FeCl_3_ at a ratio of 10:1:1 (*v*/*v*/*v*). Forty microliters of extract were mixed with 1.2 mL of FRAP reagent and incubated in the dark for 30 min at 37 °C. Absorbance was measured at 593 nm, and results were expressed as µmol Fe^2+^ per gram of extract.

### 4.7. Statistical Analysis

Results were expressed as mean values ± standard deviation of three independent biological replicates per treatment. Before the analysis, homogeneity of variance was checked using Levene’s test. Treatment effects were evaluated using one-way analysis of variance, followed by Tukey’s post hoc test at *p* < 0.05. In addition, relationships among variables were assessed through Pearson’s correlation analysis. Statistical analyses were carried out using STATGRAPHICS Centurion XVI.I and GraphPad Prism 8.0.

## 5. Conclusions

This study explored the use of LED light as a physical elicitation strategy to improve the production of high-value rosemary cell biomass. Our findings suggest that the simultaneous application of blue, red, and far-red light may generate an interactive spectral effect, potentially involving blue- and red/far-red-light perception systems. This spectral combination was associated with a significant increase in the accumulation of rosmarinic and salvianic acids, suggesting a favorable modulation of phenolic metabolism. The improved phenolic profile was accompanied by higher antioxidant capacity, as evidenced by increased ABTS^•+^, DPPH^•^, and FRAP activities. Since rosemary is a useful source of natural antioxidant compounds, including extracts authorized as food antioxidants (E392) in the EU, cultivating cell suspension cultures under optimized LED spectra may offer a sustainable, scalable, and consistent alternative to traditional field cultivation. This approach could contribute to the development of controlled production systems for rosemary-derived bioactive compounds with potential applications in food, nutraceutical, and cosmetic sectors. Future research should focus on transcriptomic profiling of critical biosynthetic genes (including PAL, TAT, RAS), conducting enzymatic kinetic assays, and scaling processes in bioreactor systems to enhance industrial implementation.

## Figures and Tables

**Figure 1:**
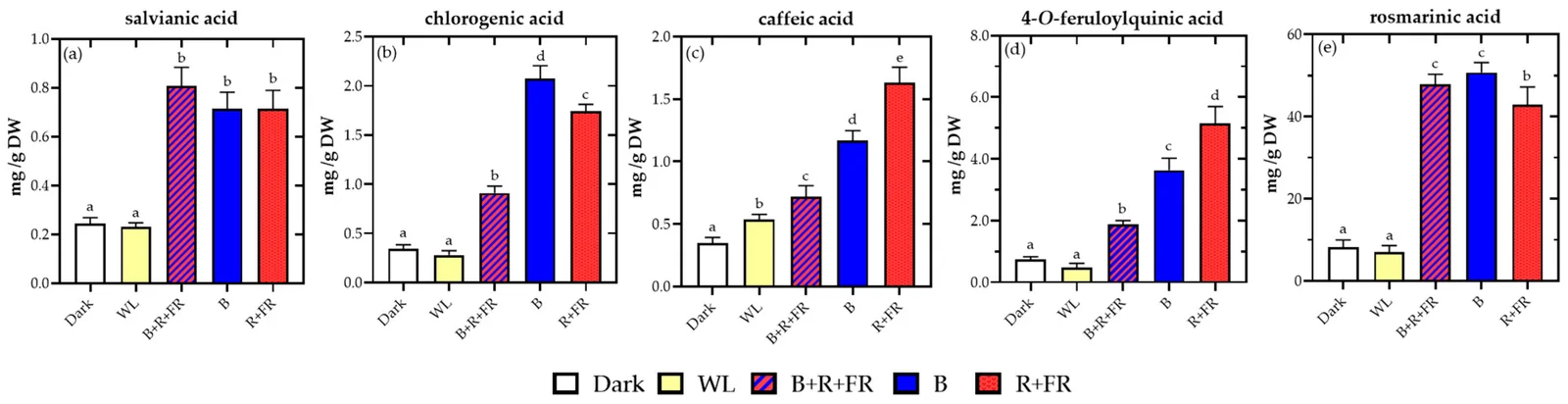
Content of phenolic compounds in cell suspension cultures of rosemary under different light treatments: (**a**) salvianic, (**b**) chlorogenic, (**c**) caffeic, (**d**) 4-*O*-feruloylquinic, and (**e**) rosmarinic acids. Results are expressed as mg g^−1^ DW, where DW refers to the dry weight of the lyophilized cell biomass used for extraction. Different letters indicate significant differences in compound content between treatments (*p* < 0.05). Abbreviations: WL, white light; B+R+FR, blue, red, and far-red light at a ratio of 25:40:35; B, blue light; and R+FR, red and far-red light at a ratio of 55:45.

**Figure 2:**
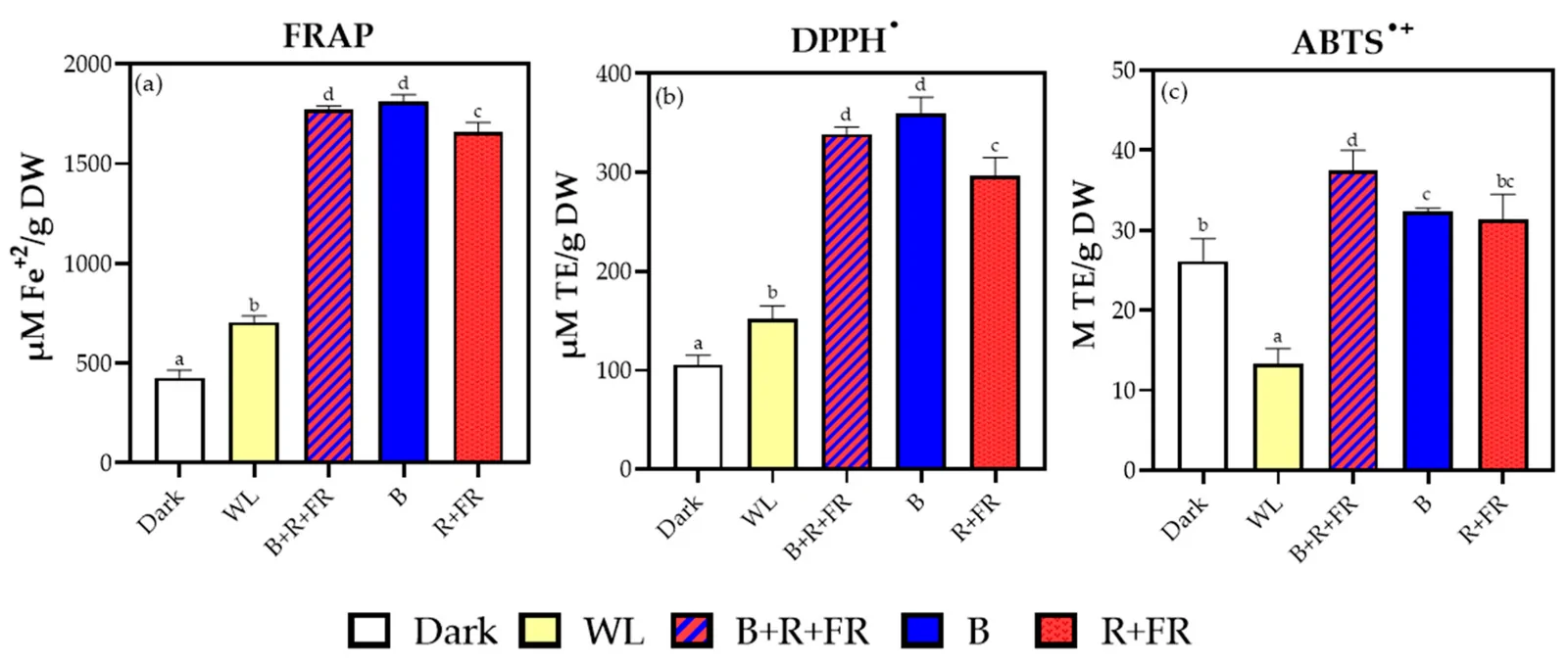
Antioxidant capacity of the extracts determined by (**a**) FRAP, (**b**) DPPH^•^, and (**c**) ABTS^•+^ assays. Antioxidant activities are expressed as µmol Trolox equivalents g^−1^ DW for DPPH and ABTS, and as µmol Fe^2+^ equivalents g^−1^ DW for FRAP. DW refers to the dry weight of the lyophilized cell biomass used for extraction. Different letters indicate significant differences in antioxidant activity between treatments (*p* < 0.05).

**Figure 3:**
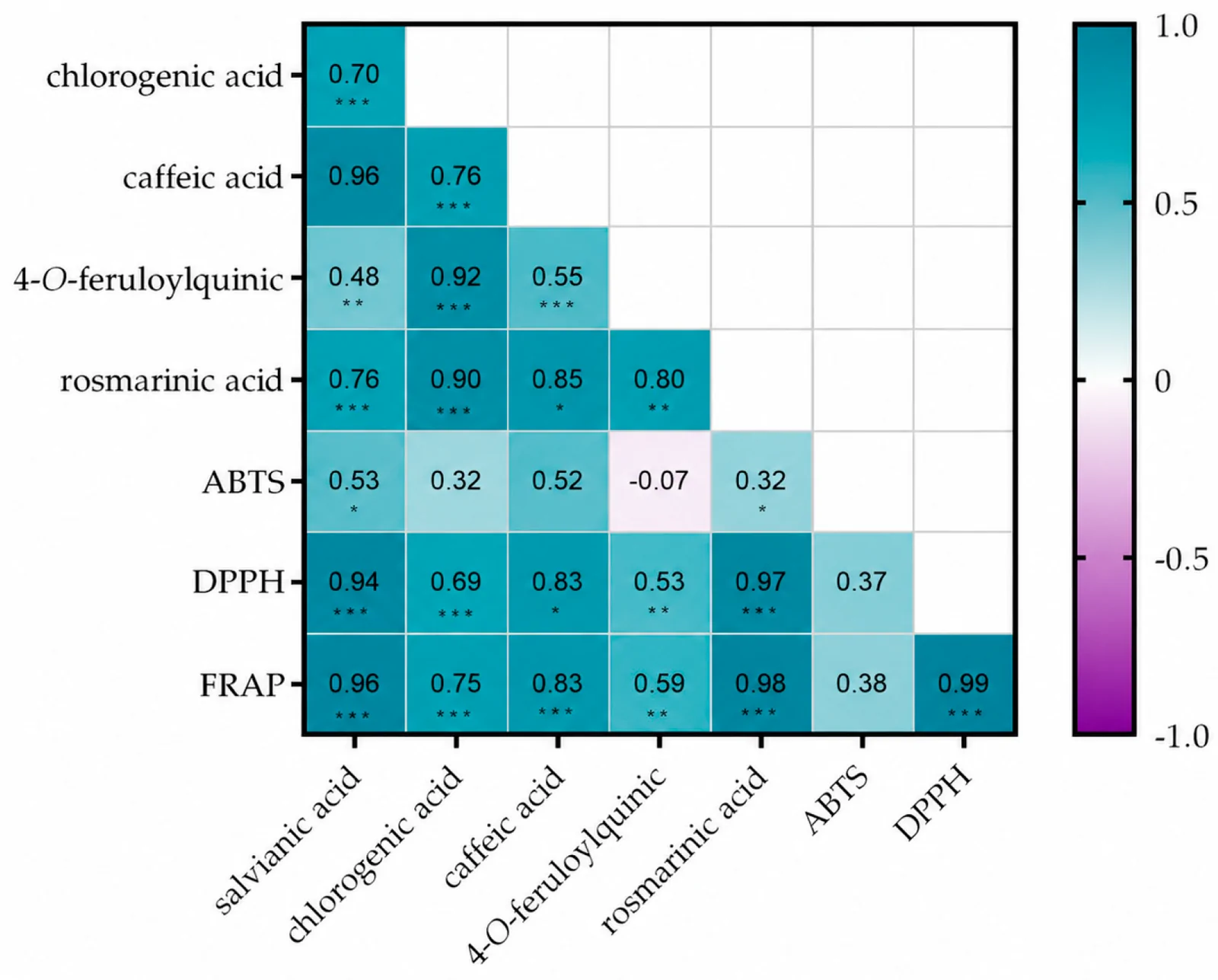
Pearson correlation heatmap showing relationships among phenolic compounds and antioxidant activity parameters in the extracts. Correlation coefficients (r) are displayed within each cell. Color intensity indicates the strength and direction of the correlation, ranging from −1 (purple, negative correlation) to 1 (turquoise, positive correlation). Asterisks indicate statistically significant correlation coefficients (* *p* < 0.05, ** *p* < 0.01, and *** *p* < 0.001).

**Figure 4:**
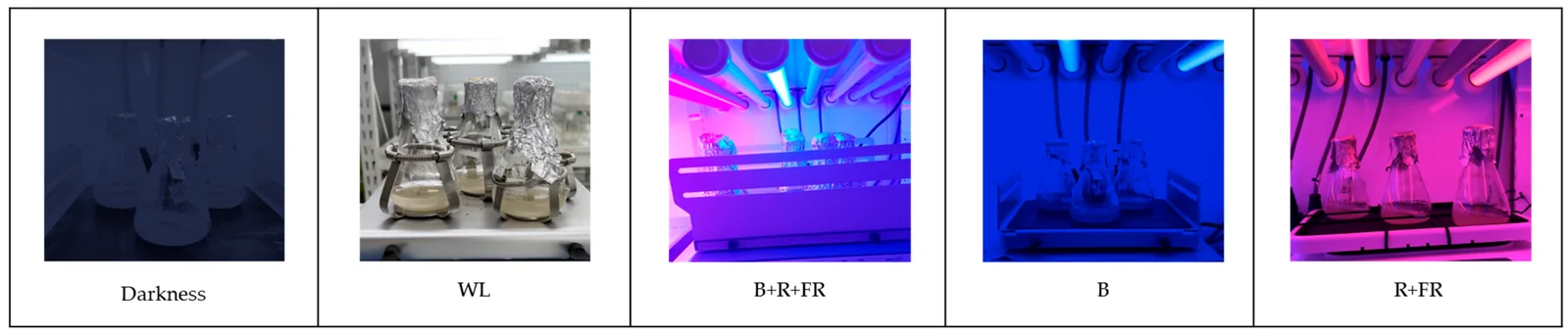
LED light treatments applied to the *Salvia rosmarinus* suspension culture. Abbreviations: WL, white light; B+R+FR, blue, red, and far-red light at a ratio of 25:40:35; B, blue light; and R+FR, red and far-red light at a ratio of 55:45.

**Table 1:** Growth parameters of rosemary cell suspension cultures under different light treatments.

Growth Parameter	Treatment				
	Dark	WL	B+R+FR	B	R+FR
Packed cell volume (%)	43.3 ± 2.9 ^ab^	45.0 ± 5.0 ^ab^	37.0 ± 2.6 ^a^	65.0 ± 5.0 ^c^	46.7 ± 5.8 ^b^
Biomass (g L^−1^)	9.7 ± 0.8 ^b^	7.5 ± 0.9 ^a^	9.3 ± 0.8 ^b^	7.6 ± 0.6 ^a^	12.5 ± 0.5 ^c^
pH	5.7 ± 0.2 ^a^	6.1 ± 0.1 ^b^	6.4 ± 0.3 ^b^	6.2 ± 0.2 ^b^	6.2 ± 0.1 ^b^
Brix levels (°Bx)	0.8 ± 0.3 ^a^	0.9 ± 0.1 ^ab^	1.1 ± 0.1 ^b^	0.8 ± 0.1 ^a^	0.9 ± 0.1 ^ab^

The results are expressed as the mean ± SD. Values are presented as mean ± SD of three independent biological replicates. Different lowercase letters within each row indicate significant differences among treatments according to Tukey’s test (*p* < 0.05). Abbreviations: WL, white light; B+R+FR, blue, red, and far-red light at a ratio of 25:40:35; B, blue light; and R+FR, red and far-red light at a ratio of 55:45.

## Data Availability

All data are available within this article.

## Supplementary Materials

The following supporting information can be downloaded at: https://www.mdpi.com/article/10.3390/plants15213322/s1, Figure S1: Photograph showing the macroscopic appearance of the cell suspension of *Salvia rosmarinus* Spenn; Figure S2: Profiles of cell growth during cultivation of *Salvia rosmarinus* Spenn. suspension cells in a 250 mL Erlenmeyer flask without elicitation. Presented data are mean values ± standard deviation (n = 3); Table S1: Initial Brix (°Bx) measurements for the culture of *Salvia rosmarinus* Spenn; Table S2: Standards used for identifying and quantifying main phenolic compounds in the cell suspension of *Salvia rosmarinus* Spenn.

## Abbreviations

The following abbreviations are used in this manuscript:

ABTS^•+^	2,2-azinobis-(3-ethylbenzothiazoline-6-sulphonic acid)
B	blue light
B+R+FR	blue, red, and far-red light combination
COP1	CONSTITUTIVE PHOTOMORPHOGENIC 1
DAD	diode array detector
DPPH^•^	2,2′-diphenyl-1-picrylhydrazyl
DWE	dry weight of the extract
FRAP	ferric reducing antioxidant power
HPLC	high-performance liquid chromatography
HY5	ELONGATED HYPOCOTYL 5 transcription factor
LED	light-emitting diode
PAL	phenylalanine ammonia-lyase enzyme
PCV	packed cell volume
R+FR	red and far-red light
TAT	tyrosine aminotransferase
WL	white light
°Bx	degrees Brix

## References

[1] Bianchin M., Pereira D., Almeida J.d.F., de Moura C., Pinheiro R.S., Heldt L.F.S., Haminiuk C.W.I., Carpes S.T. (2020). Antioxidant Properties of Lyophilized Rosemary and Sage Extracts and Its Effect to Prevent Lipid Oxidation in Poultry Pátê. Molecules.

[2] Barreto Peixoto J.A., Álvarez-Rivera G., Alves R.C., Costa A.S.G., Machado S., Cifuentes A., Ibáñez E., Oliveira M.B.P.P. (2021). Comprehensive Phenolic and Free Amino Acid Analysis of Rosemary Infusions: Influence on the Antioxidant Potential. Antioxidants.

[3] Al-Shammari S.H., Aboud K.H. (2026). Phenolic Compounds in Rosemary (*Rosmarinus officinalis* L.): Scientific Review. Eur. J. Ecol. Biol. Agric..

[4] European Commission (2012). Commission Regulation (EU) No 231/2012 of 9 March 2012 Laying Down Specifications for Food Additives Listed in Annexes II and III to Regulation (EC) No 1333/2008 of the European Parliament and of the Council (Text with EEA Relevance).

[5] Ortiz de Elguea-Culebras G., Melero-Bravo E., Ferrando-Beneyto T., Jordán M.J., Cáceres-Cevallos G., Sánchez-Vioque R. (2024). Comprehensive Study on the Potential of Domesticated Clones of Rosemary (*Salvia rosmarinus* Spenn.): Implications for Large-Scale Production and Waste Recovery in the Development of Plant-Based Agrochemicals. Agriculture.

[6] Hashim M., Ahmad B., Drouet S., Hano C., Abbasi B.H., Anjum S. (2021). Comparative Effects of Different Light Sources on the Production of Key Secondary Metabolites in Plants in Vitro Cultures. Plants.

[7] Lim W.H., Khaw M.L., Yungeree O., Hew W.H., Parab A.R., Chew B.L., Wahyuni D.K., Subramaniam S. (2025). Effects of LEDs, Macronutrients and Culture Conditions on Biomass and Artemisinin Production Using *Artemisia annua* L. Suspension Cultures. Biotechnol. Prog..

[8] Kulak M. (2020). Recurrent Drought Stress Effects on Essential Oil Profile of Lamiaceae Plants: An Approach Regarding Stress Memory. Ind. Crops Prod..

[9] Yesil-Celiktas O., Nartop P., Gurel A., Bedir E., Vardar-Sukan F. (2007). Determination of Phenolic Content and Antioxidant Activity of Extracts Obtained from Rosmarinus Officinalis’ Calli. J. Plant Physiol..

[10] Caruso J.L., Callahan J., Dechant C., Jayasimhulu K., Winget G.D. (2000). Carnosic Acid in Green Callus and Regenerated Shoots of *Rosmarinus officinalis*. Plant Cell Rep..

[11] Boix Y.F., Arruda R.C.O., Defaveri A.C.A., Sato A., Lage C.L.S., Victório C.P. (2013). Callus in *Rosmarinus officinalis* L. (Lamiaceae): A Morphoanatomical, Histochemical and Volatile Analysis. Plant Biosyst..

[12] Khan T., Abbasi B.H., Khan M.A. (2018). The Interplay between Light, Plant Growth Regulators and Elicitors on Growth and Secondary Metabolism in Cell Cultures of *Fagonia indica*. J. Photochem. Photobiol. B.

[13] Narayani M., Srivastava S. (2017). Elicitation: A Stimulation of Stress in in Vitro Plant Cell/Tissue Cultures for Enhancement of Secondary Metabolite Production. Phytochem. Rev..

[14] Murthy H.N., Joseph K.S., Paek K.Y., Park S.Y. (2024). Light as an Elicitor for Enhanced Production of Secondary Metabolites in Plant Cell, Tissue, and Organ Cultures. Plant Growth Regul..

[15] Zhao Z., Liu S., Yun C., Liu J., Yao L., Wang H. (2024). Melatonin Alleviates UV-B Stress and Enhances Phenolic Biosynthesis in Rosemary (*Rosmarinus officinalis*) Callus. Physiol. Plant..

[16] Maham N., Chamani E., Mohebodini M., Tariverdizadeh N. (2025). Optimizing Biosynthesis: The Role of LED Light Spectra in Regulating Phenolic and Flavonoid Accumulation in *Matricaria chamomilla* L. Root Cultures. BMC Plant Biol..

[17] Ptak A., Szewczyk A., Simlat M., Pawłowska B., Warchoł M. (2024). LED Light Improves Shoot Multiplication, Steviol Glycosides and Phenolic Compounds Biosynthesis in *Stevia rebaudiana* Bertoni In Vitro Culture. Sci. Rep..

[18] Jung W.S., Chung I.M., Hwang M.H., Kim S.H., Yu C.Y., Ghimire B.K. (2021). Application of Light-Emitting Diodes for Improving the Nutritional Quality and Bioactive Compound Levels of Some Crops and Medicinal Plants. Molecules.

[19] Cioć M., Szopa A., Prokopiuk B., Pawłowska B., Łopusiewicz Ł. (2025). Influence of LED Light Spectra on Morphogenesis, Secondary Metabolite Production and Antioxidant Potential in *Eucomis autumnalis* Cultured In Vitro. Agronomy.

[20] Nadeem M., Abbasi B.H., Younas M., Ahmad W., Zahir A., Hano C. (2019). LED-Enhanced Biosynthesis of Biologically Active Ingredients in Callus Cultures of *Ocimum basilicum*. J. Photochem. Photobiol. B.

[21] Fazal H., Abbasi B.H., Ahmad N., Ali S.S., Akbar F., Kanwal F. (2016). Correlation of Different Spectral Lights with Biomass Accumulation and Production of Antioxidant Secondary Metabolites in Callus Cultures of Medicinally Important *Prunella vulgaris* L.. J. Photochem. Photobiol. B.

[22] Kawka B., Kwiecień I., Ekiert H. (2017). Influence of Culture Medium Composition and Light Conditions on the Accumulation of Bioactive Compounds in Shoot Cultures of *Scutellaria lateriflora* L. (American Skullcap) Grown In Vitro. Appl. Biochem. Biotechnol..

[23] Gallego A., Imseng N., Bonfill M., Cusido R.M., Palazon J., Eibl R., Moyano E. (2015). Development of a Hazel Cell Culture-Based Paclitaxel and Baccatin III Production Process on a Benchtop Scale. J. Biotechnol..

[24] Krzemińska M., Hnatuszko-Konka K., Weremczuk-Jeżyna I., Owczarek-Januszkiewicz A., Ejsmont W., Olszewska M.A., Grzegorczyk-Karolak I. (2023). Effect of Light Conditions on Polyphenol Production in Transformed Shoot Culture of *Salvia bulleyana* Diels. Molecules.

[25] Grzegorczyk-Karolak I., Ejsmont W., Kiss A.K., Tabaka P., Starbała W., Krzemińska M. (2024). Improvement of Bioactive Polyphenol Accumulation in Callus of *Salvia atropatana* Bunge. Molecules.

[26] Zielińska S., Dryś A., Piątczak E., Kolniak-Ostek J., Podgórska M., Oszmiański J., Matkowski A. (2019). Effect of LED Illumination and Amino Acid Supplementation on Phenolic Compounds Profile in Agastache Rugosa In Vitro Cultures. Phytochem. Lett..

[27] Kim B.R., Jeong Y.J., Kim S., Kim S.B., Lee J., Lee O.R., Kim K.D., Jeong J.C., Yang B.W., Kim C.Y. (2025). Elicitor-Mediated Enhancement of Rosmarinic Acid Biosynthesis in Cell Suspension Cultures of Lavandula Angustifolia and In Vitro Biological Activities of Cell Extracts. Plant Physiol. Biochem..

[28] Cáceres-Cevallos G., Bayo Canha A., Martínez-Conesa C., García-Aledo I., Olivares L., Quílez-Simón M., Rome-ro-Espinar P., Ros P., Jordán M. (2026). Methyl Jasmonate Elicitation as a Strategy to Improve Bioactive Compound Production in *Salvia rosmarinus* Cell Suspension Cultures. Acta Hortic..

[29] Cáceres-Cevallos G.J., Jordán M.J. (2024). Effects of LED Light on Aromatic Medicinal Plants from *Lavandula*, *Salvia*, and *Thymus* Genera: A Systematic Review. Stresses.

[30] Cáceres-Cevallos G.J., Bayo-Canha A., Quílez M., Jordán M.J. (2026). LED Light Treatments Enhance the Synthesis of Bioactive Compounds in *Salvia lavandulifolia* Vahl. Plants.

[31] Cáceres-Cevallos G.J., Quílez M., Albacete-Moreno A.A., Jordán M.J. (2024). Does High-Intensity Light Pre-Treatment Improve Drought Response in *Thymus zygis* ssp. Gracilis?. Plant Stress.

[32] Quílez M., Ferreres F., López-Miranda S., Salazar E., Jordán M.J. (2020). Seed Oil from Mediterranean Aromatic and Medicinal Plants of the Lamiaceae Family as a Source of Bioactive Components with Nutritional. Antioxidants.

[33] Farhat M.B., Chaouch-Hamada R., Sotomayor J.A., Landoulsi A., Jordán M.J. (2014). Antioxidant Potential of *Salvia officinalis* L. Residues as Affected by the Harvesting Time. Ind. Crops Prod..

[34] Szabó K., Radácsi P., Rajhárt P., Ladányi M., Németh É. (2017). Stress-Induced Changes of Growth, Yield and Bioactive Compounds in Lemon Balm Cultivars. Plant Physiol. Biochem..

