# Supplementary material for: Light Quality as a Non-Chemical Elicitor of Polyphenol Accumulation in Rosemary (Salvia rosmarinus Spenn.) Cell Suspension Cultures
Source: Plants (Basel). 2026 Oct 27;15(21):3322. doi: 10.3390/plants15213322 (PMC13765543; doi:10.3390/plants15213322)
Supplement: Supplementary file 1 [file plants-15-03322-s001.zip › plants-4623649-supplementary.pdf]

plants

Supplementary Materials

Light Quality as a Non-Chemical Elicitor of Polyphenol Accumulation in Rosemary (*Salvia rosmarinus* Spenn.) Cell Suspension Cultures

Table S1. Initial Brix (°Bx) measurements for the culture of *Salvia rosmarinus* Spenn.

Treatments	°Bx	Media
Dark	4.5<br>4.5<br>4.3	4.4
WL	4.5<br>4.6<br>4.2	4.4
B+R+FR	4.3<br>3.9<br>4.1	4.1
B	4.6<br>4.1<br>4	4.2
R+FR	4.2<br>4.2<br>3.9	4.1
	**Initial °Bx of the culture**	4.3 ± 0.2

Abbreviations: WL, white light; B+R+FR, blue, red, and far-red light at a ratio of 25:40:35; B, blue light; and R+FR, red and far-red light at a ratio of 55:45.

Table S2. Standards used for identifying and quantifying main phenolic compounds in the cell suspension of *Salvia rosmarinus* Spenn.

Phenolic compound	Equation	R^2	Straight interval (ppm)	Standard used
Salvianic acid	y = 0.1736x + 0.4855	0.9998	1.5 – 10.0	Salvianic acid
Chlorogenic acid	y = 0.0361x + 0.3339	0.9991	1.5 – 20.0	Chlorogenic acid
Caffeic acid	y = 0.0114x + 0.2945	0.9983	2.0 – 20.0	Caffeic acid
4-*O*-feruloylquinic acid	y = 0.0411x + 1.3153	0.9999	10.0 - 50.0	5-*O*-feruloylquinic acid
Rosmarinic acid	y = 0.0191x + 5.2697	0.9998	50.0 – 400.0	Rosmarinic acid

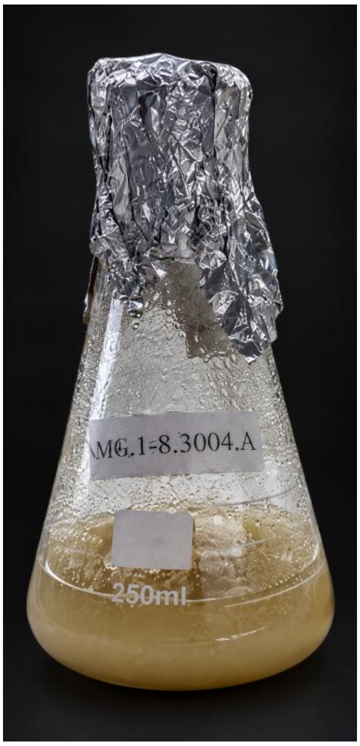

Figure S1. Photograph showing the macroscopic appearance of the cell suspension of *Salvia rosmarinus* Spenn.

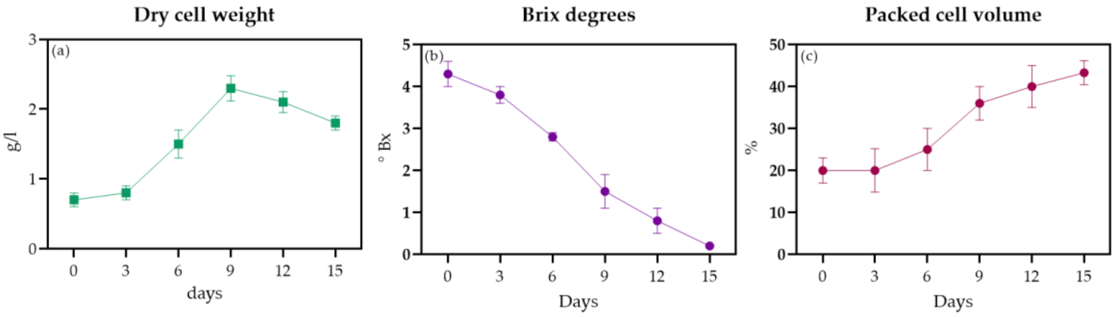

Figure S2. Profiles of cell growth during cultivation of *Salvia rosmarinus* Spenn. suspension cells in a 250 mL Erlenmeyer flask without elicitation. Presented data are mean values ± standard deviation (n = 3).
